# Individual and Community-Level Determinants of Institutional Delivery Services among Women in Bangladesh: A Cross-Sectional Study

**DOI:** 10.1155/2022/3340578

**Published:** 2022-03-26

**Authors:** Sarmistha Paul Setu, Md. Akhtarul Islam, Sk. Faijan Bin Halim

**Affiliations:** ^1^Statistics Discipline, Science Engineering & Technology School, Khulna University, Khulna 9208, Bangladesh; ^2^Economics Discipline, Khulna University, Khulna 9208, Bangladesh

## Abstract

**Background:**

Improving maternal mortality attracts considerable interest with the critical invention through institutional delivery services (IDS) in reducing maternal death during delivery and ensuring safe childbirth. The influence of both individual and community-level factors is essential to using IDS. *Problem Statement.* Maternal death may occur at any time, but delivery without designated healthcare is by far the most dangerous time for both woman and her baby. Therefore, to combat the global burden of maternal mortality, it is necessary to ensure IDS worldwide.

**Objectives:**

This study explores the current knowledge of individual and community-level covariates and examines their extent of influence on the utilization of IDS in Bangladesh.

**Methods:**

Utilizing Bangladesh Demographic and Health Survey (BDHS) data, this study has used two-level random intercept binary logistic regression, together with the average annual rate of increase (AARI) in the utilization of IDS and related variables.

**Results:**

This study found appreciable changes in seeking IDS, increases from 3.4% in 2007 to 51.9% in 2017, and half of the total deliveries (51%) took place in healthcare. About 26% of the total variation in the utilization of IDS is owing to differences across communities. Further, covariates including communities with higher educated women, higher utilization of ANC and access to media and at individual level, religion, maternal and parental education, wealth index, and mother-level factors (i.e., age at birth, BMI, occupation, ANC visit, birth order, own health care decision, pregnancy intention, and exposure to media) showed significant association with the utilization of IDS.

**Conclusion:**

This study observed the association between individual and community-level factors and IDS uptake. Thus, any future strategies must address individual level and community-level challenges and undertake a multisectoral approach to enhance the uptake of IDS.

## 1. Introduction

The utilization of maternal health care services is generating considerable interest in terms of reducing perinatal deaths and postnatal complications [1]. Despite significant improvement in the past few decades, there has been a rapid rise in maternal mortality, especially in developing countries undergoing a severe global health problem [2]. Globally, 358,000 maternal deaths take place annually, of which 95% are documented in developing countries [3]. In 2015, roughly 5,500 women in Bangladesh died from different maternal causes. Concerns have arisen owing to the adverse lifetime risk of maternal death, projected to be 1 in 240 in Bangladesh [4]. Place of delivery is considered as the most fundamental stage of the utilization of maternal health care services (i.e., antenatal care, delivery care, and postnatal care), which posits that accessibility of life-saving equipment associated with hygienic conditions is enticing widespread interest to combat the complications of delivery and thus guarantee the safety of the mother and her child [5, 6].

Given the fact that the delivery process might result in unexpected complications, therefore, it is imperative to ensure institutional, that is, public or private delivery, or skilled attendant at birth [7], a reliable approach to the safe birth outcome, clean delivery, provision of an experienced expert, and early detection of any maternal and neonatal complications [5]. In developed countries, nearly 98% of women receive antenatal care (ANC), and 94% of births are attended by experienced health personnel [8]. In contrast, several studies from 48 low- and middle-income countries have found that in sub-Saharan Africa, South Asia, and Southeast Asia, more than 70% of all births in the bottom two wealth quintiles occurred at home [9]. In Bangladesh, about 71% of total births occur at home [10], and a minority portion (around 37%) is delivered in a designated health care facility [11]. Significant downsides are probably due to the old traditional belief, shortage of healthcare professionals, poor health infrastructure, and scarce resources, especially in remote rural areas. Despite significant steps taken by the government at the grassroots level, the likelihood of service uptake is still low, especially in the rural areas with a lower concentration of healthcare professionals, poor literacy, high poverty, cheaper traditional birth attendants, and limited knowledge regarding utilization of delivery, compared to urban areas [12].

A growing body of literature has investigated the gender of household head, maternal age [13], religion [13, 14], occupation, birth order [14–16], residence [12], number of antenatal care (ANC) visits [12–14, 17, 18], knowledge towards complicacy at childbirth [2, 13, 15], household wealth status [9, 12, 13, 19], media exposure [13, 20], paternal and maternal education [9, 13, 14, 17], number of children [21, 22], and decision making on health care [23, 24] as individual level covariates that are significantly associated with the utilization of institutional delivery services. Additional studies also reported that community-level predictors, including place of residence [13, 17, 19, 22], religion, distance to the nearest health center, and community media exposure [13, 25, 26], are significantly associated with IDS.

More recent evidence carried out in Bangladesh and other settings have exemplified the predictors of IDS. Several studies have concentrated on identifying sociodemographic, physical accessibility, and individual health system covariates and confirmed significant association on the utilization of IDS [27–32]. Notwithstanding, few studies have already reported community-level factors in the utilization of IDS in South Asia and Africa [33, 34]. However, there is still considerable ambiguity in explaining the utilization of IDS at the individual and community levels in Bangladesh. Therefore, this paper has intended to outline a range of individual and community-level covariates and examine their degree of influence on the utilization of IDS in Bangladesh by using data from the 2017-18 Bangladesh Demographic and Health Survey (BDHS). Further, the findings of this current research thus seem to be warranted to inform policymakers delineating effective intervention strategies to address challenges and improve the IDS in our country.

## 2. Methods

### 2.1. Data Source and Study Design

The analysis was performed using a nationally representative survey, the 2017-18 Bangladesh Demographic and Health Survey (BDHS) [35] data. National Institute of Population Research and Training (NIPORT) of the Ministry of Health and Family Welfare (MOHFW) managed the dataset, and the survey was carried out by a Bangladeshi Research Institute named Mitra and Associates. The United States Agency provided necessary financial support for conducting this survey for International Development (USAID). We considered an enumeration area with a mean of about 120 households as a primary sampling unit (PSU).

During the survey period, a two-stage stratified sampling procedure was applied for selecting the respondents. We chose this particular apparatus because samples were stratified by geographical region and by urban or rural areas within each region. Initially, the smallest administrative units were labeled as enumeration areas or clusters (EAs) and selected with probability proportional to their size. Later on, households were selected from each EA. We considered a weighted sample of 4842 individual women who had given birth to at least one living child in the five-year period prior to the study. In addition, we considered 675 clusters (weighted) as community characteristics. The final analysis contained only the initial live birth for women who had more than one living child.

### 2.2. Outcome Variable

The dependent variable for this study was the places where the women gave birth. We categorized these delivery places into two different groups: home delivery and institutional delivery. If a woman gave birth in her own home or other's home, it was considered home delivery, and giving birth in a public, private, or nongovernmental organization was considered institutional delivery. Here, we considered the dependent variable a binary variable labeled as 1 for institutional delivery and 0 for home delivery.

### 2.3. Explanatory Variables

We considered individual and community-level factors as independent variables for performing multilevel analysis. We included age of respondent (up to 25 years, above 25 years), respondent age at first birth (less than or equal to 18, greater than or equal to 19), religion (Muslim, non-Muslim), wealth index poor (poorer and poorest), middle, rich (richer and richest), maternal education level (up to the primary, at least secondary), paternal education level (up to the primary, at least secondary), respondent's BMI (normal, overweight, or underweight), respondent's ANC visits (no visit, at least eight visits, and more than eight visits), health care decision (respondent alone or jointly, others), birth order (first birth, more than one birth), pregnancy intention (intended, unintended), respondent's employment status (working, not working), and media exposure (no, yes) as individual level factors associated with delivery place [13, 35, 36]. In this study, we considered five community-level factors, namely, community maternal education (whether more than 50% of respondents belong to the cluster were less educated (up to primary) or more educated (at least secondary)), community media exposure (whether or not more than 50% respondents of the cluster had access to media), community wealth quintile (whether or not more than 50% respondents of the cluster were in the top three wealth quintiles), community ANC utilization (whether or not more than 50% respondents of the cluster had at least 8 ANC visits), and types of place of residence (rural, urban) [13].

### 2.4. Statistical Analysis

We then performed rate of change analysis for utilization of IDS with the help of four successive BDHS data conducted in 2007, 2011, 2014, and 2017-18. In order to perform this analysis, we calculated the average rate of increase, which can be written as

(1) $$\begin{array}{c} Y_{\left(k+n\right)}=Y_{k}^{*}{\left(1+p\right)}^{n}, \end{array}$$

where *Y*_*k*_ = utilization of IDS of any particular year, *p* = rate of the yearly change, *n* = years between two studies, and *Y*_*k*+*n*_ = utilization of the (*k* + *n*)th year, which was taken from the available information in the UNICEF technical note [37], which was then modified. AARI is a geometric ratio that provides a constant rate of change during the study period. It can be interpreted as the average percent by which institutional delivery rates increased each year. We used Excel version 10.0 for calculating the values of AARI.

We used multilevel logistic regression analysis to determine individual and community-level factors associated with IDS. Here two-level multivariable multilevel logistic regression was applied. Individual-level determinants (women) were nested within the community-level determinants (clusters) in which they live. Four models with necessary variables of interest were constructed in STATA version 16.0 with xtmelogit command. At first, we constructed Model 0: the intercept-only model (null model) that contains only random intercept to determine intraclass correlation (ICC) and to test the random variation of the null model. We then constructed Model 1 by taking the explanatory variables in Model 0, which determined the effects of individual level characteristics. After that, Model 3 was constructed by taking only the community-level variables to assess the impact of community-level features. Finally, we performed the final model, Model 4. Model 4 contained both individual level factors and community-level factors. We estimated the effects of individual level and community-level factors associated with institutional delivery in terms of odds ratios with their *P* values and 95% confidence interval. Random effects were expressed in terms of Intracluster Correlation (ICC), the log-likelihood, and the Akaike Information Criterion (AIC) of the models, which were estimated to assess the fitness of the model relative to the other models [36]. We then fitted the data into the model [38, 39]. Let a binary response variable *Y*_*ij*_ be “Delivery place” (1 if women *i* in community *j* takes institutional delivery service, otherwise 0). Then, the two-level random intercept binary logistic regression model considering women at level 1 and communities (PSU) at level 2 can be written as follows:

(2) $$\begin{array}{c} \text{logit}\left(\Pi_{ij}\right)=\log\left(\frac{\Pi_{ij}}{1-\Pi_{ij}}\right)=\beta_{0j}+\sum_{k=1}^{m}\beta_{k}X_{ijk};\;i=1,2,\ldots ,n_{j},\;j=1,2,\ldots ,d,\\ \text{With }\beta_{0j}=\beta_{0}+\mu_{0j};\;\mu_{0j}∼ii\;dN\left(0,\sigma_{\mu 0}^{2}\right), \end{array}$$

where *π*_*ij*_ = Pr (*Y*_*ij*_ = 1) is the probability that the woman *i* in community *j* takes institutional delivery services, *X*_*ijk*_ is the values of *m* explanatory variables for women *i* in community *j*, *β*_*k*_ is a vector of regression coefficients to be estimated, and *β*_0_ is a fixed component. *μ*_0*j*_ is the random error at the community level.

### 2.5. Ethical Issues

We used the nationally representative dataset BDHS 2017-18 for this study. It was conducted under the authority of the National Institute of Population Research and Training (NIPORT) of the Ministry of Health and Family Welfare (MOHFW). So, it was not essential to receive ethical approval for this study.

## 3. Results

About 4842 women who have given birth to a child were included in this study to find out the linked factors to the usage of IDS. The results of this study exhibited a significant difference in receiving IDS between different categories of variables: religion, maternal and parental educational level, household wealth status, mother's age at delivery, BMI level, mother's employment status, ANC visit, order of birth, intention of pregnancy, own health care decision, and media exposure status.

The proportion of women using institutional delivery by individual level characteristics was shown in Table 1. Among 4842 respondents, 2445 respondents (50.50%) delivered their child to healthcare. The utilization of IDS services was elevated among women aged up to 25 years, higher educated non-Muslim women. Maternal Education Level showed a significant effect on receiving IDS. Higher educated parents (at least secondary) had a higher rate to deliver their child to an institution. Among mothers having at least secondary education, 60.5% took institutional delivery facilities. Household wealth had a significant impact on the utilization of institutional delivery. Wealthy individuals had a higher rate of institutional delivery than poor- and middle-class individuals. A large proportion of mothers of this study who crossed 18 years at their age of childbirth had a greater chance of getting institutional delivery than mothers aged below 19 years. Among mothers with overweight or underweight BMI levels, 56.7% gave birth in an institution instead of home. Women who reported more than 8 ANC visits had higher odds of receiving IDS than women who did not receive antenatal care and 1 to 8 ANC visits. Women with more than one birth had a higher rate of taking IDS. Women who wanted to be pregnant when they met their pregnancy had a higher chance to take institutional delivery than women who did not want to be pregnant at that time. Here, among 4443 respondents who had any intention of being pregnant, about 2312 respondents (52.0%) had institutional delivery.

The results showed that women belonging to urban areas had a higher likelihood of taking IDS than those belonging to rural areas. Women from communities with an elevated part of well-off households, higher educated mothers, mothers having ANC utilization, and women having media access had an advanced rate of institutional delivery than their low concentration parts.

### 3.1. Change in Institutional Delivery over Time

Table 2 shows the average annual rate of increase in the utilization of IDS in Bangladesh from 2007 to 2017. In 2007, about 24.5% of women used healthcare services for giving birth child. In 2011, that percentage decreased in the amount 24.1%. In 2014, it became 40.1%, and finally, in 2017, about half (50.5%) women took IDS. The institutional delivery rate increased roughly by 26% from 2007 to 2017. From 2007 to 2017, the annual rate of increase of IDS was highest among non-Muslim women (35.39%) than Muslim women in Bangladesh. Women having the lowest educational level (up to primary) reported a higher AARI of IDS (21.06%). Similarly, a lower educated husband reported a higher AARI (17.69%). We observed an overall increase in the utilization of IDS within various quintiles of household wealth from 2007 to 2017. The AARI was highest among women in middle-class households (36.11%), and it was lowest among wealthy families (13.67%). The AARI was similar across two different categories of the age of the mother at first birth. This study found that the AARI of IDS utilization increases apparently with the increase in receiving ANC. The total AARI of IDS utilization was found to be maximal (21.22%) among individuals who had more than 8 ANC visits and lowest (10.21%) among women having 1 to 8 ANC visits. The AARI of institutional delivery was reported as almost similar across two different categories of mother's BMI. Women having no media access showed an increase in AARI (36.04%) of IDS utilization compared to those who had access to media. Women with first-order birth had a higher rise in AARI (17.63%) of IDS utilization than women with higher-order (more than one birth) birth. Working women reported a higher AARI of IDS (19.04%) than nonworking women. We found a maximum AARI value (20.11%) of IDS utilization within the women who did not make decisions about their health.

### 3.2. Measure of Variation (Random Effects)

To check whether the data of the current study validate the choice to evaluate the randomness at community level, a null model (intercept-only model) was established, as shown in Table 3, column 1 (Model 0). Model 0 included only the dependent variables along with aggregation of community level. The results of Model 0 illustrated that there was a meaningful variation in the probability of using IDS among women who come from different communities (variance = 1.157, 95% CI = (0.936, 1.43), and *P* value = .000). The ICC value of the intercept-only model suggested that 26% of the total variation in receiving IDS was recognized to imparities between the communities.

### 3.3. Measures of Associations (Fixed Effects)

After creating an intercept-only model and determining the number of differences in receiving IDS among communities, we created Model 1 by adding only individual level variables in Model 0 shown in Table 3. The result of Model 1 showed that religion, maternal and parental education, wealth index, media exposures, respondent's BMI, pregnancy intention, birth order, respondent's current age, respondent's age at birth, ANC visits, and health care decision had a statistically significant association with delivering a child at healthcare. The value of intraclass correlation in Model 1 pointed that 11.40% of the diversities in the utilization of IDS were responsible for the differences across communities. We then created Model 2 by taking just community-level variables in Model 0, and eventually, Model 3 was established by taking individuallevel variables and community-level variables represented in Table 3. The value of intraclass correlation from Model 2 indicated that distinctions between communities were responsible for 10.90% of the differences in respondents' IDS utilization.

### 3.4. Effect of Individual-Level Variables on Choosing IDS

Respondents having at least secondary education had 1.5 times (AOR = 1.59, 95% CI = 1.35–1.88) elevated odds of giving birth in an institution than the respondents having up to the primary level of education. Like respondent's education, their husbands having at least secondary education had 1.4 times (AOR = 1.44, 95% CI = 1.23–1.68) elevated chance to take their wives in an institution for delivering than those having up to the primary level of education. The odds of delivery in the institution among respondents of age above 25 years old was about 1.2 times (AOR = 1.16, 95% CI = 0.973–1.38) higher than those up to 25 years old. We found that respondents belonging to the highest wealth quintile had a 92% elevated (AOR = 1.92, 95% CI = 1.57–2.35) probability of receiving IDS than the respondents belonging to the lowest wealth quintile. Muslim respondents had a lower likelihood of delivering a child in an institution than non-Muslim religions. Respondents having access to media had a 24% greater chance (AOR = 1.24, 95% CI = 1.05–1.46) of delivering a child at an institution than respondents with no access to media. The result showed that respondents who visited healthcare and took medical facilities at least eight times (ANC) had 3.9 times (AOR = 3.86, 95% CI = 2.72–5.47) elevated likelihood of delivering a child in an institution than the respondents who did not visit healthcare for antenatal care. Similarly, respondents who visited healthcare and took ANC facilities more than eight times had 7.4 times (AOR = 7.43, 95% CI = 4.70–11.76) greater probability of giving birth at an institution compared to respondents who did not visit healthcare or did not take any facilities. Respondents with overweight or underweight BMI had a 33% higher (AOR = 1.33, 95% CI = 1.16–1.54) likelihood of receiving IDS compared to respondents having normal BMI. We noticed that respondents who had the right to decide about their health care had more opportunities (AOR = 1.18, 95% CI = 1.01–1.38) to deliver a child at an institution than the respondents who had no rights. Women having intentional pregnancy had a 55% higher (AOR = 1.55, 95% CI = 1.18–2.04) probability of delivering a child in an institution than women having unintentional pregnancy.

### 3.5. Effect of Community-Level Variables on Choosing IDS

In addition, this study aimed to determine whether the characteristics of the community in which people live have an impact on the preference of a place of delivery. We found that respondents from urban areas had a 12% elevated probability of giving birth at an institution compared to the respondents belonging to a rural community (AOR = 1.12, 95% CI = 0.922–1.36). Respondents belonging to communities with high concentration of education (AOR = 1.31, 95% CI = 1.08–1.61), high ANC utilization (AOR = 1.48, 95% CI = 1.24–1.76), affluent households (AOR = 1.17, 95% CI = 0.927–1.47), and communities with access to media (AOR = 1.44, 95% CI = 1.14–1.82) had a significant association with the utilization of IDS.

The estimated value of ICC of the final model indicated that 9.2% of the variation in mother's institutional delivery utilization was responsible for the distinctions between communities. The value of the proportion of change in variance (PCV) of Model 3 suggested that both the individual and community-level variables explained 71.2% of the disparities in the utilization of IDS through communities.

## 4. Discussion

Different countries have attempted to fulfill the Sustainable Development Goal (SDG) framework, mainly to accelerate the pace of reducing maternal mortality within 2030. Maternal deaths occur before and after childbirth due to various gynecological complications [40], obstetric complications, and other health complications [41–43]. Therefore, access to high-quality care during pregnancy and during and after childbirth is required for healthy childbirth and reducing maternal death.

There exists a comparative high AARI of receiving IDS among occupied respondents, up to 25-year-old respondents, non-Muslim respondents, respondents who visited more than eight visits, respondents having first birth, respondents whose pregnancy was unwanted, respondents who had no contribution in their own healthcare decision, and respondents having no access to media. About half of the deliveries took place in health care facilities, and the AARI for IDS from 2007 to 2017 was 7.50. Several studies used AARI to explore the average annual rate of increase for different selected variables [44–46].

Our results identified several individuallevel predictors along with community-level predictors related to the usage of IDS such as religion, maternal education level at individual and community level, respondent's age at first birth, respondent's ANC visit status at individual and community level, respondent's BMI, paternal education, respondent's employment status, pregnancy intention, wealth index at individual and community-level, birth order, health care decision, type of place of residence, access to media at individual and community level. Among all of five community-level predictors, community with respondents having a higher degree of education, community with respondents having access to media, and community with respondents having higher ANC visits showed a significant impact on the utilization of IDS in Bangladesh.

This study found that both the individual and community-level variables significantly impact using IDS. This result is analogous to the previous studies [23, 36, 47–49]. We found a positive association between high maternal and paternal education with the utilization of IDS at both individual and community level, which supports the findings of different studies on the usage of IDS taking place in Bangladesh [13, 34, 50–52] as well as in other countries [35, 53–55]. Education makes an individual more cautious about her health issues and helps to overcome the common prejudices that interrupt the utilization of IDS. Higher educated women and their husbands better perceive the symptoms of pregnancy complications and the benefits of medical attention for childbirth. As a result, they choose IDS for safe birth [50, 56–59] to avoid any undesirable outcomes. Since women's education plays a vital role in the utilization of IDS, investment in men's as well as women's educational programs will reduce maternal mortality.

The findings of this study suggested a positive association between communities with wealthier households and the use of IDS. Individuals from the most affluent families had a higher probability of delivering a child to an institution than individuals who belong to the poorest households. These results coincide with the existing literature [59–64]. This might be the fact that the most impoverished families spend most of their income on food and cannot afford education and medical care. Although the government has taken different inspiring activities, higher transportation costs, low daily wages, and the high fees of delivery services are responsible for the low rate of IDS utilization for individuals belonging to the poorest households [53]. Hence, the pragmatic policy at the national level can have a beneficial effect on increasing the use of specialized IDS and reducing maternal death through alleviating poverty in the country.

In this study, access to media was also an essential factor of IDS usage. Individuals having access to media (reading newspaper or magazines, listening to the radio, and watching television) had a higher chance of giving birth in healthcare than their nonexposure counterparts, which follows the results of the previous studies [65–67]. Individuals having access to media across the communities also had a higher likelihood of utilizing IDS [35]. The simple fact may explain how individuals can quickly get various health messages, information about risk factors of maternal health, and promotion of institutional delivery through multiple media programs on TV and radio. Or, by reading the newspaper, they can gather this valuable information [36]. The outcomes of this study suggest that broadcasting the importance of adopting IDS on television, radio, and newspapers may help achieve maternal and child health-related goals in Bangladesh. Consistent with earlier studies [13, 50, 68], religion was another factor in this study responsible for the variation in choosing a particular delivery place. This analysis observed that Muslim women had less likelihood of delivering a child at an institution compared to non-Muslim women [50, 69]. Women of Islam may take less assistance from health care for their religious beliefs and cultural behavior. And their husbands also prohibit them from going to male doctors of healthcare [70, 71].

Outcomes of this study confirmed that individuals having at least eight antenatal care visits (ANC) and more than 8 ANC visits had an elevated probability of taking IDS compared to the women who did not take ANC. Here a certain number of ANC was taken for this study according to new WHO ANC guidelines [72] and a survey of ANC of Benin [73]. Community ANC was also a significant predictor of institutional delivery. Women belonging to a high concentration of ANC showed availability or at least 8 ANC visits in these communities. Like other research, the outcomes of this study found that attendance of ANC increases the chance of IDS [50, 74–76]. Generally, an ANC visit makes the pregnant woman and her family conscious of the dangerous signs of labor and pregnancy complications to come [77]. As a matter of fact, alleviating maternal deaths and safe childbirth can be ensured by encouraging women to utilize IDS and providing them with the necessary information on ANC.

In this study, child's birth order was also an essential variable for utilization of IDS. That is, the odds of receiving IDS reduced with arise in the order of birth. Women of first-order birth had a higher prospect of taking IDS than individuals with second- or higher-order birth, which is consistent with the findings of several previous studies [35, 50, 69, 78]. A potential explanation is that women having more kids take delivery as a standard action and develop the self-assurance to deliver the child at home [53]. The outcomes of this study suggested that the mother's age during childbirth had a significant impact on the utilization of IDS. Older women (greater than or equal to nineteen) had higher odds of delivering in an institution than their younger counterparts (below nineteen), which is similar to the existing studies [13, 78, 79]. This may be due to the increase in various health-related complications during pregnancy with age (high blood pressure, gestational diabetes, etc.). For this reason, older women may deliver in an institution more than younger women [63]. We found that women living in urban areas had a higher probability of having IDS than women in rural areas, which supports the results of the previous studies [13, 34, 35, 80]. The most common reason for not choosing IDS among rural women may be high transportation costs, long distance between home and health care, and unconsciousness of rural women about the availability of maternal facilities [58].

It is a matter of surprise that our research revealed that working women had a lower chance of delivering a child in intuitions compared to their nonworking counterparts. This is consistent with the study [69]. For time constraints, working women who cannot take proper ANC visits consequently do not use IDS [81]. Pregnancy intention was another significant predictor responsible for utilizing IDS. This study found that individuals with unintended pregnancies had a lower probability of taking IDS than individuals with intended pregnancies. This result is similar to the previous study [69, 82]. This may be because women who do not want to be pregnant may attempt to terminate their pregnancies at the very beginning period or want to hide from other people. As a result, their probability of taking the facility from health care is lower [68]. However, this is in agreement with the finding of the studies [63, 83], where the own healthcare decision of women was found to play an essential role in utilizing IDS. Women who had the right to decide on their healthcare had a higher likelihood of using IDS than those who had not the right to make a decision. This may be explained as women in many countries do not have the right to decide on their healthcare but are bound to seek consent from their husbands or other family members. As a result, they cannot express their intention to utilize healthcare facilities [81, 83, 84].

## 5. Strengths and Limitations

The analysis utilized four consecutive nationally representative Bangladesh demographic and health survey 2007 to 2017-18 datasets. The research explored several potential variables that showed association and influence to utilize IDS among Bangladeshi women. The methods and analyzing tools used here are well known and have widely used implementations. The average annual rate of increase (AARI) indicated the increase and decrease rate of IDS for different selected variables. We have found several community-level factors affecting the utilization of IDS that can help the policymaker make appropriate policies to increase the access and use of IDS.

Inevitably, there were some discrepancies in this study because we were unable to include various potential predictors related to the usage of IDS, such as husband's occupation, distance to a health facility, awareness of community clinic, and timing to first antenatal visit. Unfortunately, it was not possible to rule out some unintended bias due to unavailability and missing information of related variables in this study. Further, we divided the religion into “Muslim” and “non-Muslim” categories and determined the odds of other religions relative to Muslim. This has limited the scope of generalizing other religions in the analysis. Above all, establishing any causal relationships was not possible for the cross-sectional structure of the present study.

## 6. Conclusions and Recommendations

The study results are promising for the stakeholders and stress the importance of taking planning intervention to stimulate IDS in Bangladesh. Instead of investigating individual level covariates only, this study also devises community-level determinants that significantly influence IDS utilization. The most intriguing correlation between community-level predictors and the uptake of IDS validates the need to emphasize community empowerment and focus on underprivileged communities. The evidence also implies the need for tackling individual level challenges to enhance the uptake of IDS. Thus, increasing IDS utilization can be made by improving community education, increasing the number of ANC visits, community exposure to mass media, and other health services. Maternal and paternal education, wealth status, and place of residence are contributing factors to accessing IDS, strengthening the necessity to combat the current disparity that persists in these sectors. Thus, promoting a multisectoral approach would be beneficial in improving maternal health on one side and reducing barriers in accessing IDS on the other.

## Figures and Tables

**Table 1:** Bivariate analysis of institutional delivery services by individual-level factors.

Individual-level characteristics	Institutional delivery		*χ* ^2^	*P* value
	No (%)	Yes (%)		
*Respondent's current age*			5.156	0.025
Up to 25 years	1372 (48.1%)	1478 (51.9%)		
Above 25 years	1025 (51.5%)	967 (48.5%)		
*Religion of the respondent*			24.29	0.000
Muslim	2240 (50.6%)	2188 (49.4%)		
Non-Muslim	157 (37.9%)	257 (62.1%)		
*Maternal education level*			376.140	0.000
Up to primary	1135 (68.9%)	512 (31.1%)		
At least secondary	1262 (39.5%)	1933 (60.5%)		
Paternal education level			373.777	0.000
Up to primary	1478 (64.1%)	829 (35.9%)		
At least secondary	919 (36.3%)	1616 (63.7%)		
*Wealth index*			603.380	0.000
Poor	1391 (68.5%)	641 (31.5%)		
Middle	437 (49.8%)	440 (50.2%)		
Rich	569 (29.4%)	1364 (70.6%)		
*Respondent's age at first birth*			169.450	0.000
≤18 years	1563 (57.8%)	1140 (42.2%)		
≥19 years	834 (39.0%)	1305 (61.0%)		
*Respondent's BMI*			46.398	0.000
Normal	1591 (53.4%)	1390 (46.6%)		
Overweight or underweight	806 (43.3%)	1055 (56.7%)		
*Respondent's employment status*			98.046	0.000
Working	1119 (58.3%)	801 (41.7%)		
Not working	1278 (43.7%)	1644 (56.3%)		
*Antenatal care visits*			323.571	0.000
No visit	342 (88.1%)	46 (11.9%)		
At least 8 visits	1981 (47.9%)	2155 (52.1%)		
More than 8 visits	74 (23.3%)	244 (76.7%)		
*Birth order*			153.398	0.000
More than one birth	1700 (56.4%)	1312 (43.6%)		
First birth	697 (38.1%)	1133 (61.9%)		
*Health care decision*			4.147	0.023
Respondent alone or jointly	1713 (48.6%)	1811 (51.4%)		
Others	684 (51.9%)	634 (48.1%)		
*Pregnancy intention*			51.236	0.000
Unintended	266 (66.7%)	133 (33.3%)		
Intended	2131 (48.0%)	2312 (52.0%)		
*Media exposure*			342.427	0.000
No	1166 (67.3%)	566 (32.7%)		
Yes	1231 (39.6%)	1879 (60.4%)		
*Community-level characteristics*				
*Place of residence*			163.238	0.000
Rural	1789 (56.1%)	1399 (43.9%)		
Urban	608 (36.8%)	1046 (63.2%)		
*Community maternal education concentration*			335.634	0.000
Low	1410 (63.9%)	797 (36.1%)		
High	987 (37.5%)	1648 (62.5%)		
*Community wealth concentration*			350.981	0.000
Poor	1232 (66.6%)	617 (33.4%)		
Rich	1165 (38.9%)	1828 (61.1%)		
*Community exposure to media*			364.163	0.000
No	1028 (70.4%)	433 (29.6%)		
Yes	1369 (40.5%)	2012 (59.5%)		
*Community ANC utilization*			279.859	0.000
Low	1490 (61.5%)	932 (38.5%)		
High	907 (37.5%)	1513 (62.5%)		

**Table 2:** Average annual rate of increase (AARI) in the utilization of IDS services in Bangladesh, 2007–2017.

Variable	2017		2014		2011		2007	
	Institutional delivery (%)	Percent AARI (2007–2017)	Institutional delivery (%)	Percent AARI (2014–2017)	Institutional delivery (%)	Percent AARI (2011–2014)	Institutional delivery (%)	Percent AARI (total, 2007–2011)
*Respondent's current age*								
Up to 25 years	51.9	31.33	24.1	29.14	3.2	96.01	3.4	−1.50
Above 25 years	48.5	8.68	15.9	45.03	9.9	17.11	21.1	−17.23
*Religion of the respondent*								
Muslim	49.4	8.67	36.3	10.82	3.2	124.69	21.5	−37.88
Non-Muslim	62.1	35.39	3.7	156.03	20.9	−43.85	3	62.46
*Maternal education level*								
Up to primary	31.1	21.06	9.7	47.46	5.5	20.82	4.6	4.57
At least secondary	60.5	11.76	30.3	25.92	18.6	17.66	19.9	−1.67
*Parental education level*								
Up to primary	35.9	17.69	13.9	30.79	7.2	24.52	6.1	4.23
At least secondary	63.7	12.64	26.1	32.34	16.9	15.59	18.4	−2.10
*Wealth index*								
Poor	31.5	27.84	8.0	57.91	3.3	34.34	2.7	5.14
Middle	50.2	36.11	7.1	91.93	3.5	26.59	2.3	11.07
Rich	70.6	13.67	25.0	41.35	17.2	13.28	19.6	−3.21
*Respondent's age at first birth*								
≤18 years	42.2	15.72	19.3	29.79	10.7	21.73	9.8	2.22
≥19 years	61.0	15.29	20.8	43.14	13.4	15.79	14.7	−2.29
*Respondent's BMI*								
Normal	46.6	15.74	22.7	27.09	11.1	26.93	10.8	0.69
Overweight or underweight	56.7	15.26	17.4	48.26	13.0	10.21	13.7	−1.30
*Respondent's employment status*								
Working	41.7	19.04	—	—	0.1	−100	7.3	−65.79
Not working	56.3	12.59	—	—	24.6	−100	17.2	9.36
*Antenatal care visits*								
No visit	11.9	20.79	2.3	72.96	2.1	3.08	1.8	3.93
At least 8 visits	52.1	10.21	35.7	13.43	20.0	21.31	19.7	0.38
More than 8 visits	76.7	21.22	2.1	231.79	2.0	1.64	11.2	−34.99
*Birth order*								
More than one birth	43.6	13.49	20.2	29.23	11.4	21.01	12.3	−1.88
First birth	61.9	17.63	19.9	45.98	12.7	16.15	12.2	1.01
*Health care decision*								
Respondent alone or jointly	51.4	11.83	—	—	16.1	—	16.8	−1.06
Others	48.1	20.11	—	—	8.0	—	7.7	0.96
*Pregnancy intention*								
Unintended	33.3	25.27	37.3	−3.71	—	—	3.5	—
Intended	52.0	9.49	7.5	90.68	—	—	21.0	—
*Media exposure*								
No	32.7	36.04	8.1	102.54	3.3	34.89	3.1	1.58
Yes	60.4	6.30	32.0	7.36	20.8	15.44	21.5	−0.82

Respondent's employment status and health care decision data were unavailable for 2014, and pregnancy intention data were unavailable for 2011.

**Table 3:** Multilevel logistic regression analysis of individual- and community-level factors associated with institutional delivery services.

Characteristics of fixed effects	Model 0^a^	Model 1^b^		Model 2^c^		Model 3^c^	
		AOR (95% CI)	*P* value	AOR (95% CI)	*P* value	AOR (95% CI)	*P* value
*Respondent's current age*							
Up to 25 years ^ref^		1				1	
Above 25 years		1.20 (1.01–1.42)	0.046			1.16 (0.973–1.38)	0.096
*Religion of the respondent*							
Muslim ^ref^		1				1	
Non-Muslim		1.59 (1.21–2.10)	0.001			1.58 (1.20–2.07)	0.001
*Maternal education level*							
Up to primary ^ref^		1				1	
At least secondary		1.68 (1.43–1.98)	0.000			1.59 (1.35–1.88)	0.000
*Parental education level*							
Up to primary ^ref^		1				1	
At least secondary		1.45 (1.24–1.69)	0.000			1.44 (1.23–1.68)	0.000
*Wealth index*							
Poor ^ref^		1				1	
Middle		1.35 (1.11–1.64)	0.003			1.21 (0.99–1.47)	0.063
Rich		2.46 (2.04–2.96)	0.000			1.92 (1.57–2.35)	0.000
*Respondent's age at first birth*							
≤18 years ^ref^		1				1	
≥19 years		1.35 (1.17–1.57)	0.000			1.37 (1.18–1.59)	0.000
*Respondent's BMI*							
Normal ^ref^		1				1	
Overweight or underweight		1.36 (1.18–1.56)	0.000			1.33 (1.16–1.54)	0.000
*Respondent's employment status*							
Working ^ref^		1				1	
Not working		1.37 (1.18–1.59)	0.000			1.34 (1.15–1.56)	0.000
*Antenatal care visits*							
No visit ^ref^		1				1	
At least eight visits		4.28 (3.02–6.07)	0.000			3.86 (2.72–5.47)	0.000
More than eight visits		9.31 (5.89–14.71)	0.000			7.43 (4.70–11.76)	0.000
*Birth order*							
More than one birth^ref^		1				1	
First birth		1.69 (1.43–2.01)	0.000			1.68 (1.41–1.99)	0.000
*Heath care decision*							
Others ^ref^		1				1	
Respondent alone or jointly		1.21 (1.04–1.42)	0.015			1.18 (1.01–1.38)	0.035
*Pregnancy intention*							
Unintended ^ref^		1				1	
Intended		1.51 (1.15–1.98)	0.003			1.55 (1.18–2.04)	0.002
*Media exposure*							
No ^ref^		1				1	
Yes		1.46 (1.25–1.72)	0.000			1.24 (1.05–1.46)	0.012

*Community-level factors*							
*Place of residence*							
Rural ^ref^				1		1	
Urban				1.35 (1.11–1.62)	0.002	1.12 (0.922–1.36)	0.252
*Community maternal education concentration*							
Low ^ref^				1		1	
High				1.71 (1.41–2.08)	0.000	1.31 (1.08–1.61)	0.007
*Community wealth concentration*							
Poor ^ref^				1		1	
Rich				1.54 (1.23–1.91)	0.000	1.17 (0.927–1.47)	0.187
*Community exposure to media*							
No^ref^				1		1	
Yes				1.76 (1.41–2.21)	0.000	1.44 (1.14–1.82)	0.002
*Community ANC utilization*							
Low^ref^				1		1	
High				1.91 (1.61-2.27)	0.000	1.48 (1.24–1.76)	0.000
*Measures of variation for a random effect*							
Community-level variance (SE)	1.16^*∗*^(0.125)	0.423^*∗*^(.076)		0.404^*∗*^(.068)		0.334^*∗*^(.068)	
Explained variation (PCV)	Reference	63.53		65.17		71.21	
ICC (%)	26.00	11.40		10.90		9.20	
*Model fitness*							
Log Likelihood	−3158.09	−2731.90		−2992.53		−2690.70	
AIC	6320.19	5497.80		5999.06		5425.39	

Ref = reference, OR = odds ratio, CI = confidence interval, ^a^intercept or null model, ^b^model includes only individual-level predictors, ^c^includes only community-level predictors, ^d^full model includes significant individual and community-level predictors, ^i^compared with the intercept or null model, and *∗*significance of the null model at 1% level of significance.

## Data Availability

Bangladesh Demographic and Health Survey (BDHS) has been used, which is available from the corresponding author upon request. Data are available at https://dhsprogram.com/.

## Abbreviations

MM: Maternal mortality
IDS: Institutional delivery services
BMI: Body mass index
ANC: Antenatal care
Ref: Reference
OR: Odds ratio
CI: Confidence interval
AARI: Average annual rate of increase.
